# MicroRNA-92a-3p enhances functional recovery and suppresses apoptosis after spinal cord injury via targeting phosphatase and tensin homolog

**DOI:** 10.1042/BSR20192743

**Published:** 2020-05-04

**Authors:** Shaoxuan He, Zhihua Wang, Yunxuan Li, Junjie Dong, Dong Xiang, Lirong Ren, Limin Guo, Jun Shu

**Affiliations:** 1 Department of Emergency Surgery, Second Affiliated Hospital of Kunming Medical University; 2 Department of Traumatology, Second Affiliated Hospital of Kunming Medical University; 3 Department of Orthopedics, First Affiliated Hospital of Kunming Medical University

**Keywords:** AKT/mTOR pathway, MicroRNA-92a-3p, Phosphatase and tensin homolog, Spinal cord injury

## Abstract

Spinal cord injury (SCI) is a neurological disease commonly caused by traumatic events on spinal cords. MiRNA-92a-3p is reported to be down-regulated after SCI. Our study investigated the effects of up-regulated miR-92a-3p on SCI and the underlying mechanisms. SCI mice model was established to evaluate the functional recovery of hindlimbs of mice through open-field locomotion and scored by Basso, Beattie, and Bresnahan (BBB) locomotion scale. Apoptosis of spinal cord cells was determined by flow cytometry. The effects of miR-92a-3p on SCI were detected by intrathecally injecting miR-92a-3p agomiR (agomiR-92) into the mice prior to the establishment of SCI. Phosphatase and tensin homolog (PTEN) was predicted as a target of miR-29a-3p by TargetScan. We further assessed the effects of agomiR-92 or/and overexpressed PTEN on apoptosis rates and apoptotic protein expressions in SCI mice. Moreover, the activation of protein kinase B (AKT)/mammalian target of rapamycin (mTOR) signaling was determined by Western blot. The results showed that compared with the sham-operated mice, SCI mice had much lower BBB scores, and theapoptosis rate of spinal cord cells was significantly increased. After SCI, the expression of miR-92a-3p was down-regulated, and increased expression of miR-92a-3p induced by agomiR-92 further significantly increased the BBB score and decreased apoptosis. PTEN was specifically targeted by miR-92a-3p. In addition, the phosphorylation levels of Akt and mTOR were up-regulated under the treatment of agomiR-92. Our data demonstrated that the neuroprotective effects of miR-92a-3p on spinal cord safter SCI were highly associated with the activation of the PTEN/AKT/mTOR pathway.

## Introduction

Annually more than 250000 people suffer from spinal cord injury (SCI) worldwide, and the incidence has been increasing [1]. SCI is a neurological disease that is commonly caused by traumatic events on the spinal cords [2,3]. Based on previous results derived from studies using animal models of SCI, the pathogenesis of SCI can be divided into the primary mechanical injury, and secondary damage that are characterized by various pathophysiologic mechanisms including oxidative stress [4], mitochondrial dysfunction [5] and inflammation [6]. The prognosis of SCI is significantly poor and could lead to permanent movement disorders and cognition impairment or even paralysis [7]. Although the underlying pathophysiology of SCI has been widely investigated, currently there is no effective treatment for SCI, due to abnormal responses of cells and tissues to the injury [8].

Phosphatase and tensin homolog (PTEN) has potent ability in regulating cell growth and proliferation, and is identified as a promising tumor suppressor [9–11]. In addition to anti-tumor effect, PTEN also plays a particularly important role in regulating axon regeneration after SCI [12]. Neuronal apoptosis in the central nervous system (CNS) is a major cause of the secondary damage after SCI, and could result in the exacerbation of axon injury and limit restorative processes [13,14]. The promoting effects of PTEN on axon regeneration was initially reported in 2008, when Park et al. [15] found that the conditional deletion of PTEN could reactivate the mammalian target of rapamycin (mTOR) pathway and subsequent axon regeneration in wild-type adult mice. Similarly, Gutilla et al. [12] showed that early postnatal genetic deletion of PTEN allows the regeneration of axons after SCI in adult mice. These data suggest that PTEN is potentially an effective therapeutic target in SCI treatment.

MiRNAs are a class of small and non-coding regulatory RNAs and can negatively regulate the post-transcriptional expressions of target genes through binding to the 3′-untranslated regions (3′-UTRs) of their mRNAs [16,17]. Approximately 30% mRNAs are regulated by miRNAs which allow mRNAs to widely participate in the regulation of various cellular bioprocesses, including cell proliferation, differentiation, apoptosis and metastasis [18,19]. Some miRNAs are recently demonstrated to be involved in the pathogenesis of secondary SCI [20], and could possibly become the potential targets for SCI treatment. For instance, the elevation of miR-21 contributes to the repair of injured spinal cords through suppressing programmed cell death 4 (PDCD4) [21]; miR-544a is down-regulated after SCI, and up-regulating miR-544a levels could effectively attenuate inflammation and promote the recovery of spinal cord through inhibiting neurogenic differentiation 4 (NEUROD4) expression [3]. Moreover, miR-92a-3p is found to be significantly down-regulated after SCI [22], however, its functions in SCI pathology remains to be determined.

## Materials and methods

### Animals

All female C57BL/6 mice (25–30 g, 8–10 weeks) were purchased from Shanghai SLAC Laboratory Animal Co., Ltd. (Shanghai, China). The animals were housed in an animal experimental center at 22 ± 2°C in 40–60% relative humidity under a 12-h light/dark cycle. The animal experiments and protocols were approved by the Animal Care and Use Committee of the Second Affiliated Hospital of Kunming Medical University and conducted in accordance with Institutional Animal Care Guidelines of the hospital.

### Spinal cord contusion injury

The mice were anesthetized using 2.5% isoflurane gas in oxygen and subjected to laminectomy at T8–T9 levels to expose the spinal cords. For SCI construction, the spinous processes of T7 and T10 were clamped to stabilize the spine, and the spinal cord was moderately compressed for 15 s under anesthesia as described previously [23]. The mice in the Sham group were subjected to laminectomy alone but without contusion injury. After the surgery, 200 μl of 0.9% saline solution were injected intraperitoneally into the mice to supply body liquid. The bladder was manually emptied twice daily.

### Experimental design

#### Experiment 1

The mice were randomized into Sham and SCI groups to assess the establishment of SCI model and cell apoptosis of spinal cord. The locomotor activity of the mice was evaluated according to Basso, Beattie, and Bresnahan (BBB) score methods at 1, 3 and 7 days after the surgery and then once a week for 4 weeks. Three days after the surgery, the mice in Sham and SCI groups were killed by 50 mg/kg Nembutal, followed by 30 ml PBS cardiac perfusion. Spinal cords were subsequently exposed, and a 10-mm long segment of the cords centered at the injury epicenter was collected for the detection and analysis of cell apoptosis and expression.

#### Experiment 2

In order to explore the functions of miR-92a-3p in SCI, miR-92a-3p agomiR (agomiR-92) and agomiR control (agomiR-ctrl) were obtained from Shanghai GenePharma Co., Ltd. (Shanghai, China). The mice were assigned to three groups, namely, the Sham, SCI + agomiR-ctrl, and SCI + agomiR-92 groups. The changes of miR-92a-3p expression were detected to evaluate the effects of agomiR-92 on miR-92a-3p expression in the mice under the intrathecal treatment using agomiR-92 (20 nmol/ml). The mice in the Sham group received a laminectomy served as the control, while those in the SCI + agomiR-ctrl and SCI + agomiR-92 groups were subjected to SCI induction for 3 days after the intrathecal treatment of agomiR-92 and agomiR-ctrl. Subsequently, locomotor activity was evaluated by BBB score at 1, 3 and 7 days after the surgery and then once a week for 4 weeks after agomir-494 treatment. Three days after the agomiR-92 treatment, spinal cords centered at the injury epicenter were collected for subsequent experiments.

#### Experiment 3

MiR-92a-3p could target PTEN, thus, the functions of miR-92a-3p/PTEN axis in the progression of SCI were further evaluated. Overexpressed PTEN vector and agomiR-92 were intrathecally and synchronously injected into the mice. Overexpressed PTEN and negative control (NC) vectors were obtained from GenePharma. The mice were randomized into four groups, namely, agomiR-ctrl + NC group (mice treated by NC vector and agomiR-ctrl before the SCI construction), agomiR-ctrl + PTEN group (mice treated by PTEN vector and agomiR-ctrl before the SCI construction), agomiR-92 + NC (mice treated by agomiR-92 and NC), and PTEN group (mice treated by PTEN before the SCI construction). Three days after the surgery, the spinal cords of each group were harvested for apoptosis detection and expression analysis.

### Behavioral assessment

The mice were subjected to open-field locomotion for evaluating their recovery of the voluntary locomotive function of hindlimbs, by using BBB locomotion scale at 1, 3 and 7 days after the surgery and then once a week for 4 weeks. Behavioral analyses were performed by two independent investigators blinded to the experimental groups. The mice were detected to eliminate baseline deficit before the SCI induction. Next, the mice were placed on the platform, and the limb exercise of hindlimbs were scored and observed, and each test lasted 4 min. The final score of each mouse was determined by the average scores from the two investigators. The scores ranged from 0 to 21 (0 indicates no movement, while 21 indicates normal movement).

### Hematoxylin and Eosin staining

The tissues were paraffin-embedded and sliced to 4–7 µm thick. Then the slices were dewaxed by xylene for 10 min and washed by ethanol (100 and 95%). The slices were dyed by Hematoxylin for 5 min and 0.5% Eosin for 1 min. Next, the slices were dehydrated by ethanol (95 and 100%) for 5 min and dewaxed by xylene for 10 min. After sealing the sliced using neutral gum, the results were observed under a DMi8 optical microscope (Leica, Germany).

### Apoptosis detection

Cell apoptosis of spinal cord tissues was determined by flow cytometry [24]. Spinal cord tissues were digested by 2.5 g/l trypsin for 10 min and then filtered through a 200-mesh sieve. The cells were collected by centrifuging at 1000 rpm for 5 min, and the supernatant was discarded. Cell apoptosis was measured using the Annexin V-FITC/propidium iodide (PI) cell apoptosis detection kit (Sigma–Aldrich, Merck KGaA, Darmstadt, Germany). Next, The cells were reseeded into 24-well plates at 1 × 10^6^ cells/well by Annexin V-FITC and PI and further incubation at 37°C for 30 min. Cell apoptosis was measured by flow cytometry (BD Biosciences, CA, U.S.A.).

### Luciferase reporter assay

TargetScan prediction detected a possible interaction site between PTEN and miR-92a-3p. MiR-92a 3p mimic and mimic control were synthesized by GenePharma. The fragment of wild-type PTEN-3′-UTR containing the putative miR-494 interaction site was cloned into the pMIR-REPORT vector (Ambion, Invitrogen, Carlsbad, CA, U.S.A.). Mutant PTEN-3′-UTR was generated using a QuikChange Site-Directed Mutagenesis Kit (Agilent Technologies, Santa Clara, CA, U.S.A.) to destroy the binding. Mutant PTEN-3′-UTR was also cloned into luciferase vector (PTEN-MUT). PTEN-MUT and PTEN-WT was respectively co-transfected with miR-144-3p mimic into the BV-2 cells (Chinese Academy of Medical Sciences, Beijing, China) using Lipofectamine 2000 (Invitrogen). After 48-h post-transfection, luciferase activity was determined by dual-luciferase reporter assay system (Promega, Madison, WI, U.S.A.).

### Quantitative real-time RT-PCR

Total RNAs were isolated from spinal cord tissues and transfected BV-2 cells for cDNA synthesis. For mRNA expression analysis, reversed transcription of RNAs was performed using cDNA synthesis kit (Invitrogen). Relative mRNA levels were measured using SYBR green master mix (Applied Biosystems, U.S.A.) on an ABI 7500 Real-Time PCR System (Applied Biosystems). The reaction condition was set at 95°C for 30 s, followed by 40 cycles at 95°C for 5 s and at 60°C for 20 s. TaqMan® microRNA reverse transcription kit (Applied Biosystems) and SYBR® Premix Ex Taq™ Kit (Takara, Dalian, China) were used to determine the relative levels of miR-29a-3p according to the manufacturer’s instructions, and then quantified by the 2^−ΔΔ*C*_T_^ method. U6 and GAPDH served as internal controls. All primers’ sequences are shown in Table 1.

**Table 1:** Primers for RT-qPCR

Gene name	Primer sequences
*miR-92a-3p*	Forward: 5′-TATTGCACTTGTCCCGGCCTG-3′
	Reverse: 5′-TGTCGTGGAGTCGGCAATTG-3′
*PTEN*	Forward: 5′-TGGGGAAGTAAGGACCAGAG-3′
	Reverse: 5′-GGCAGACCACAAACTGAGGA-3′
*U6*	Forward: 5′-CTCGCTTCGGCAGCACA-3′
	Reverse: 5′-AACGCTTCACGAATTTGCGT-3′
*GAPDH*	Forward: 5′-TGTTCCTACCCCCAATGTG-3′
	Reverse: 5′-GTGTAGCCCAAGATGCCCT-3′

### Western assay

Spinal cord tissues and transfected BV-2 cells were homogenized by lysis buffer (Beyotime Biotechnology, Shanghai, China). Protein concentration was quantified by BCA protein assay kit (Takara). A total of 15 μg proteins were isolated on 10% SDS/polyacrylamide gels and transferred to polyvinylidene difluoride (PVDF) membranes (Millipore, Bedford, U.S.A.). After blocking the membranes for 2 h, the membranes were incubated with primary antibodies overnight at 4°C, and then cultured with horseradish peroxidase-labeled secondary antibodies (1:20000, #ab205718 and #ab205719, 42 and 52 kDa, Abcam, Cambridge, MA, U.S.A.) at room temperature for 1 h at 4°C. The proteins were visualized by an enhanced chemiluminescence system (Millipore). Primary antibodies used were as follows: B-cell lymphoma-2 (Bcl-2, 1:1000, 26 kDa, #ab59348, Abcam), Bax (1:2000, 21 kDa, #ab32503, Abcam), Cleaved Caspase-3 (C caspase-3, 1:1000, 17 kDa, #ab2302, Abcam), PTEN (1:10000, 47 kDa, #ab32199, Abcam), p-protein kinase B (AKT, 1:1000, 60 kDa, #9271, CST, Danvers, MA, U.S.A.), Akt (1:1000, 60 kDa, #9272, CST), p-mTOR (1:1000, 289 kDa, #2971, CST), mTOR (1:1000, 289 kDa, #2972, CST) and GAPDH (1:20000, 36 kDa, #ab8245, Abcam).

### Statistical analysis

Data were shown as mean ± SD. Differences between the two groups were analyzed using Student’s *t* test. One-way analysis of variance (ANOVA) followed by Dunnett’s *t* test was performed for the statistical difference in the mean values among multiple groups. *P*<0.05 was considered to be statistically significant.

## Results

### The assessment of behavior and cell apoptosis of SCI mice model

**Figure 1:**
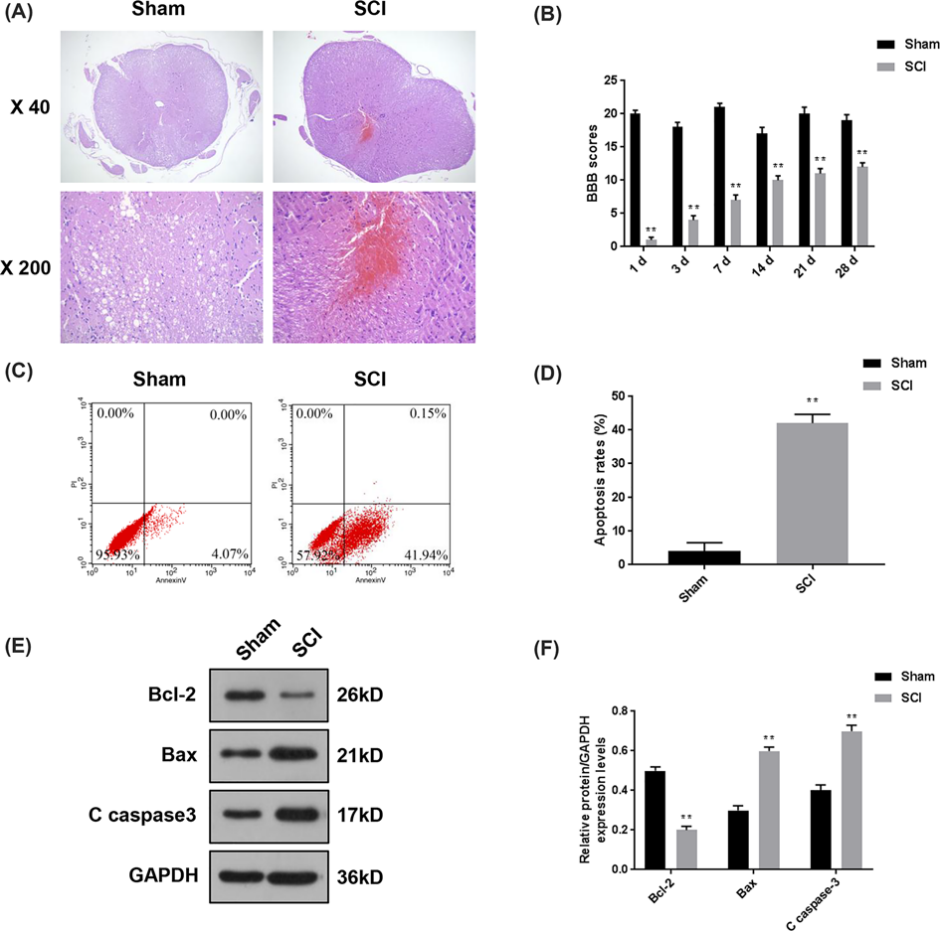
The assessment of behavior and cell apoptosis in SCI mice model In order to investigate the functions of miR-92a-3p in the progression of SCI, we established SCI mice model. (**A**) HE staining was used to ensure the successful construction of SCI model. (**B**) Open-field locomotion by BBB locomotion scale was used to evaluate the functional deficits of hind limbs of mice at 1 3, 7, 14, 21 and 28 days post-SCI. (**C,D**) The changes of apoptosis rates of spinal cord tissues were detected by flow cytometry. (**E,F**) The protein levels of several types of apoptotic indicators (Bcl-2, Bax and C caspase-3) were determined by Western blot. Each value represents mean ± SD. GAPDH served as the internal control. **P<0.01 vs. Sham.

The SCI mice model was constructed, moreover, HE staining was used to ensure the successful construction of SCI model, and open-field locomotion scored by BBB locomotion scale was performed to evaluate the functional deficits of hindlimbs of the mice after SCI. The samples of the Sham group showed clear structure after HE staining, and almost no bleeding or inflammatory cells could be found. In the SCI group, a large amount of infiltration of red blood cells and inflammatory cells were observed (Figure 1A). During open-field walking, SCI mice showed obvious paralysis of bilateral hindlimbs, however, those in the sham-operated walked normally. As listed in Figure 1B, BBB scores of SCI mice were much lower than those of the Sham group. Although the scores of SCI group increased gradually over time, there were obvious differences between SCI and Sham groups at the 28th day after the surgery (*P*<0.01). We further measured the apoptosis rates of spinal cord cells, and observed that the apoptosis rates in SCI mice were noticeably higher than that in Sham group (42.09 vs. 4.07%, *P*<0.01, Figure 1C,D). In addition, the changes in expression levels of Bcl-2, Bax and C caspase-3 further demonstrated that the spinal cord tissues of SCI mice had severe cell apoptosis (*P*<0.01, Figure 1E,F). Thus, these data suggested that the SCI mice model had been established successfully.

### Overexpressed miR-92a-3p improved BBB scores and inhibited cell apoptosis of SCI mice

**Figure 2:**
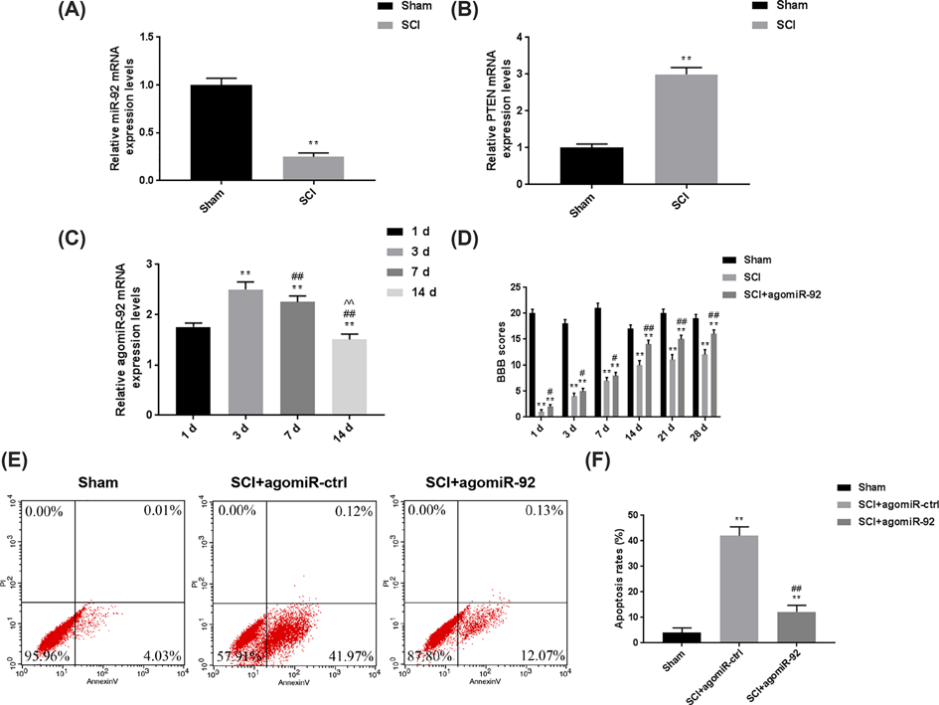
Overexpressed miR-92a-3p improved BBB scores and inhibited cell apoptosis of SCI mice In order to verify the functions of miR-92a-3p in the progression of SCI, the mice were treated intrathecally by agomiR-92 prior to the SCI surgery. (**A**) The mRNA level of miR-92a-3p in the SCI mice was compared with that in sham-operated mice. (**B**) The mRNA level of PTEN in SCI mice was compared with that in the sham-operated mice. **P<0.01 vs. Sham. (**C**) The changes of relative mRNA levels of miR-92a-3p were determined by qRT-PCR at 1, 3, 7 and 14 days after intrathecal treatment of agomiR-92. **P<0.01 vs. 1d; ^##^*P*<0.01 vs. 3d; ^^^^*P*<0.01 vs. 7d. (**D**) The motor function of SCI mice was evaluated through BBB rating scale at 1, 3, 7,14, 21 and 28 days in SCI mice model. (**E,F**) The changes in apoptosis rates of spinal cord tissues were analyzed by flow cytometry following agomiR-92 treatment. Each value represents mean ± SD. GAPDH and U6 served as the internal controls. **P<0.01 vs. Sham; ^#^*P*<0.05, ^##^*P*<0.01 vs. SCI + agomiR-ctrl. Abbreviation: qRT-PCR, quantitative real-time RT-PCR.

In order to verify the roles of miR-92a-3p after SCI, agomiR-92 was intrathecally injected into the mice prior to the surgery. The results in Figure 2A,B showed that compared with Sham group, the miR-92a-3p level in SCI mice was obviously down-regulated, while PTEN expression was significantly up-regulated (*P*<0.01). We also investigated the effects of agomiR-92 on miR-92a-3p expression in the mice by recording the changes in miR-92a-3p level during 14 days after agomiR-92 treatment. As shown in Figure 2C, agomiR-92 effectively exerted its function in SCI mice, and the mRNA level of agomiR-92 was obviously up-regulated (*P*<0.01) and peaked at day 3. We subsequently established SCI mice models 3 days after the agomir-92 treatment, and the motor function of SCI mice was evaluated by BBB scale. As shown in Figure 2D, up-regulating miR-92a-3p level could notably promote the recovery of motor functions and improve the BBB scores (*P*<0.01). Furthermore, the apoptosis rate of SCI + agomiR-92 group was greatly reduced compared with that in SCI + agomiR-ctrl group (Figure 2E,F). Taken together, overexpressed miR-92a-3p by agomiR-92 could notably promote the functional recovery and inhibit SCI-associated cell apoptosis in mice. Though it might be a limitation to not obtain immunostaining images, the mRNA levels also could reflect miR-92a-3p and PTEN levels.

### MiR-92a-3p was predicted to specifically target PTEN

**Figure 3:**
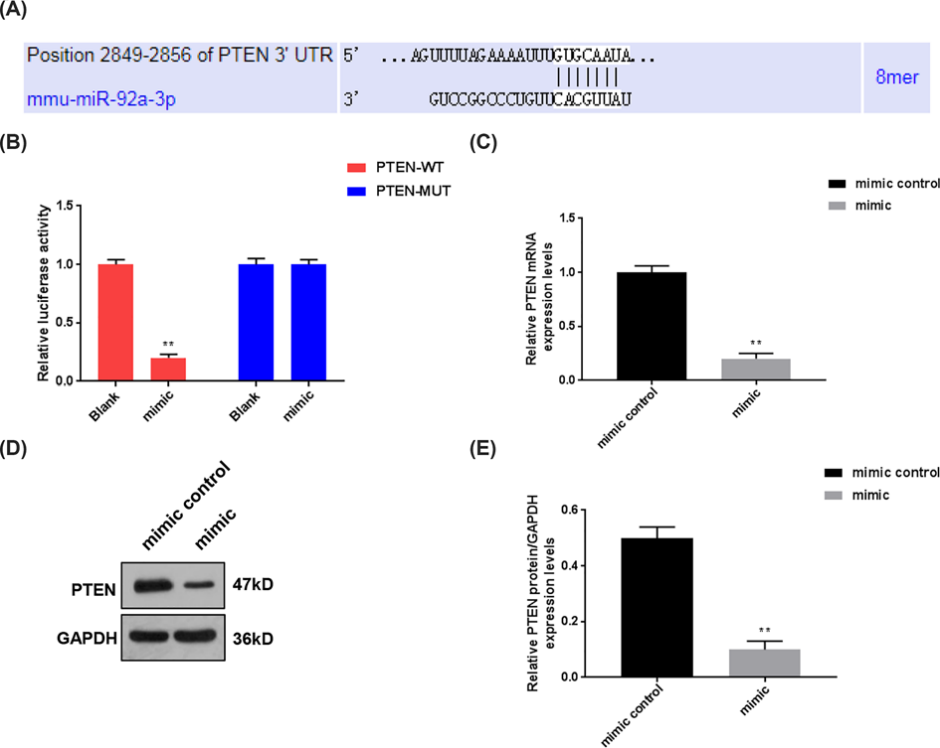
PTEN was identified as a target for miR-92a-3p To further investigate the mechanisms underlying the positive effects of miR-92a-3p on SCI progression, we screened the possible target genes for miR-92a-3p by TargetScan. (**A**) A possible binding site of miR-92a-3p was identified in the 3′-UTR of PTEN. (**B**) The dual-luciferase reporter assay was performed to verify the association between PTEN and miR-92a-3p. **P<0.01 vs. Blank. (**C**–**E**) We further measured the expressions of PTEN in BV-2 cells transfected with miR-92a-3p mimic by qRT-PCR and Western blot. Each value represents mean ± SD. GAPDH served as an internal control. **P<0.01 vs. mimic control. Abbreviation: qRT-PCR, quantitative real-time RT-PCR.

The sequence of PTEN-3′-UTR was predicted to contain a potential binding site that could be specifically targeted by miR-92a-3p (Figure 3A). As BV-2 cells share numerous features with primary microglia, the cells were selected for luciferase reporter assay to further confirm the association between PTEN and miR-92a-3p [25]. Two types of luciferase vectors containing wild-type and mutant 3′-UTR of PTEN were constructed, as shown in Figure 3B, miR-92a-3p obviously inhibited the luciferase activity of PTEN-WT vectors, but it did not affect PTEN-MUT vectors (*P*<0.01, Figure 3B). In addition, the mRNA and protein levels of PTEN were also notably inhibited by miR-92a-3p in BV-2 cells (*P*<0.01, Figure 3C–E). Thus, our data suggest that miR-92a-3p could directly regulate PTEN expression.

### Overexpressed PTEN partially reversed the reduced apoptosis rates induced by agomiR-92 in SCI mice

**Figure 4:**
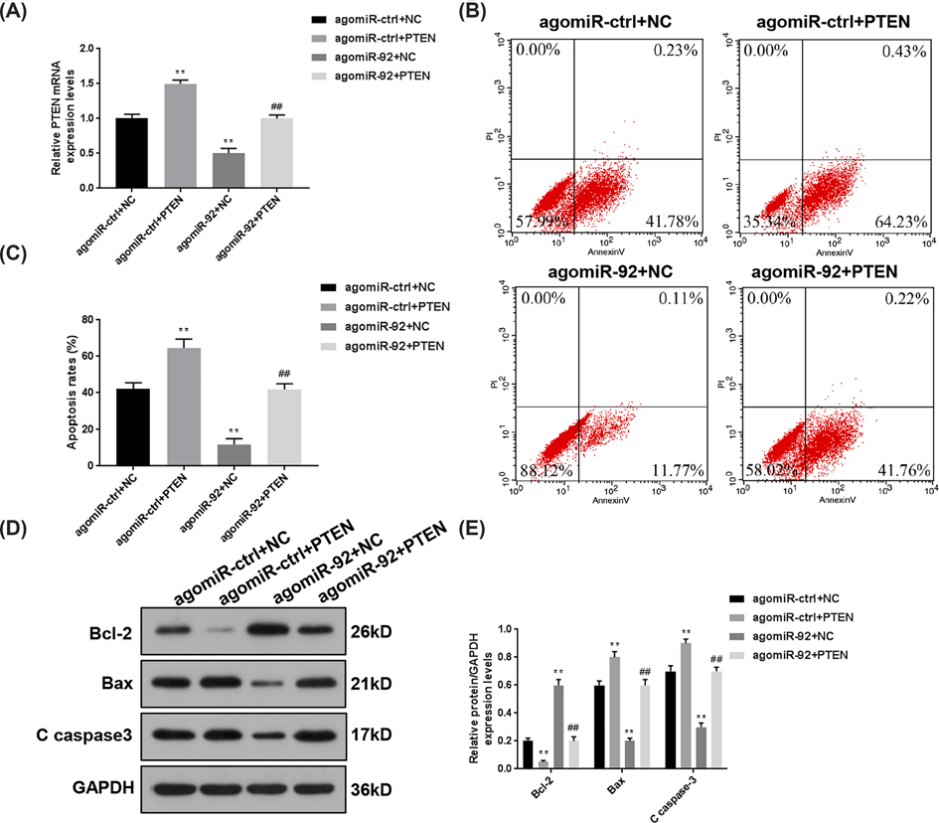
Overexpressed PTEN partially reversed the decreased apoptosis rates induced by agomiR-92 in SCI mice In order to confirm the role of miR-92a-3p/PTEN axis in the progression of SCI, the mice were intrathecally treated by both agomiR-92 and PTEN before SCI surgery. (**A**) The relative mRNA levels of PTEN were determined by qRT-PCR following SCI. (**B,C**) Flow cytometry was performed to analyze the changes in apoptosis rates in spinal cord tissues of SCI mice. (**D,E**) The protein levels of Bcl-2, Bax and C caspase-3 were determined by Western blot. Each value represents mean ± SD. GAPDH served as an internal control. **P<0.01 vs. agomiR-ctrl + NC; ^##^*P*<0.01 vs. agomiR-92 + NC. Abbreviation: qRT-PCR, quantitative real-time RT-PCR.

To confirm the role of miR-92a-3p/PTEN axis in the progression of SCI, the mice were intrathecally treated by both agomiR-92 and PTEN before the SCI surgery. We detected the expressions of PTEN in spinal cord tissues collected from every experimental group, and the data in Figure 4A demonstrated that miR-92a-3p from agomiR-92 effectively inhibited the PTEN expression. The changes in apoptosis rate of spinal cord cells were further analyzed, as shown in Figure 4B,C, the apoptosis rate was remarkably reduced from 42.01 to 64.66% by up-regulating PTEN expression in agomiR-ctrl + NC group (*P*<0.01), while the treatment of agomiR-92 alone suppressed the apoptosis rate to 11.88% (*P*<0.01). After the mice had been treated intrathecally by both agomiR-92 and PTEN, the apoptosis rate was increased to 41.98% compared with agomiR-92 + NC group (*P*<0.01). Furthermore, the changes of Bcl-2, Bax and C caspase-3 at protein level showed that the overexpression of PTEN could obviously promote the cell apoptosis in spinal cord tissues of SCI mice and partially reverse the decreased cell apoptosis induced by agomiR-92 (Figure 4D,E). It should be noted that it might be a limitation to not using immunostaining images, however, flow cytometry was also a classical method to detect the apoptosis rate.

### AgomiR-92 induced the activation of AKT/mTOR pathway through suppressing PTEN in SCI mice

**Figure 5:**
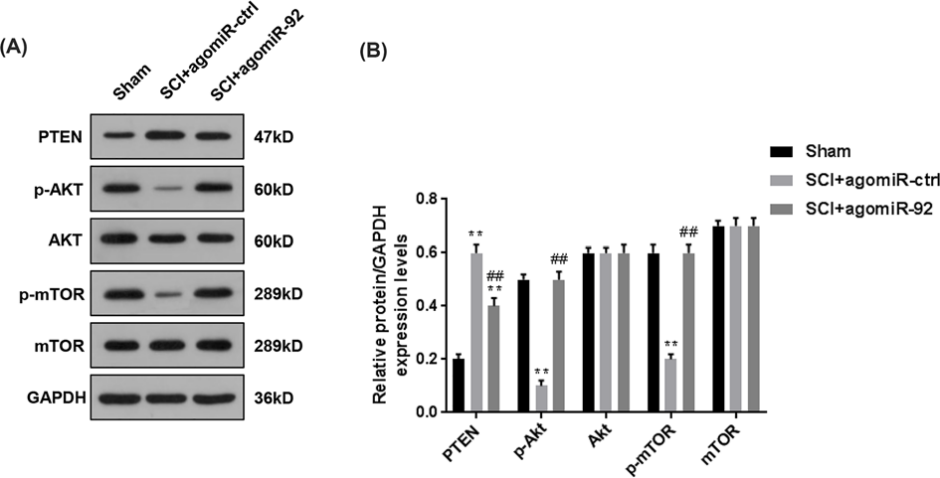
AgomiR-92 induced the activation of AKT/mTOR pathway through inhibiting PTEN in SCI mice Previous studies have demonstrated that PTEN functions as an inhibitor of AKT/mTOR pathway, therefore, we further detected the phosphorylation levels of Akt and mTOR to reflect the activation of AKT/mTOR pathway. (**A,B**) After the treatment of agomiR-92, the protein level of PTEN and the phosphorylation and protein levels of Akt and mTOR were determined by Western blot. Each value represents mean ± SD. GAPDH served as the internal control. **P<0.01 vs. Sham; ^##^*P*<0.01 vs. SCI + agomiR-ctrl.

It has previously been demonstrated that PTEN functions as an inhibitor to AKT/mTOR pathway in many cancers [10,24]. In our study, miR-92a-3p directly targeted PTEN, thus, we speculated whether miR-92a-3p contributed to the activation of AKT/mTOR pathway through targeting PTEN. Therefore, the phosphorylation and protein levels of Akt and mTOR were measured, shown in Figure 5A,B, it could be found that compared with SCI + agomiR-ctrl group, the treatment of agomiR-92 suppressed the protein level PTEN and promoted the phosphorylation levels of Akt and mTOR after SCI. However, no differences in the protein levels of Akt and mTOR were observed. Therefore, agomiR-92 could inhibit PTEN and promote the activation of AKT/mTOR pathway in SCI mice.

## Discussion

SCI is a serious CNS injury and can result in permanent movement disorders, cognition impairment and even paralysis [26]. Although it has been widely discovered that the mechanisms underlying the secondary injury of SCI involve numerous molecular and biochemical changes such as inflammatory activation, production of free radicals, axonal plasticity and neuronal cell death [27], SCI still remains one of the major challenges in clinical practice [28]. This study explored the functions of miR-92a-3p in the progression of SCI, and our data demonstrated that up-regulating miR-92a-3p level could effectively promote the recovery of motor functions and inhibit cell apoptosis of spinal cord tissues of SCI mice. In addition, PTEN was identified to be the target for miR-92a-3p. Therefore, the inhibitory effects of miR-92a-3p on cell apoptosis might be associated with the activation of the PTEN/AKT/mTOR pathway.

A series of miRNAs have been identified to be involved in the development of several SCI-associated neurological disorders [29,30]. Due to their strong regulatory abilities on gene expressions, miRNAs are involved in multiple pathophysiological processes of SCI, for instance, He et al. [31] found that reducing miR-136-5p expression could effectively suppress the production of inflammatory cytokines and chemokines, and subsequently, improve SCI via NF-κB/A20. Similarly, the transfection of miR-99b-5p inhibitor could promote neuro-regeneration in SCI mice through the activation of mTOR pathway [1]. By performing miRNA microarray analyses, miR-92a-3p has been found to be obviously down-regulated after SCI surgery [22]. Previous studies have demonstrated that miR-92a-3p is involved in many different types of diseases such as T-cell acute lymphoblastic leukemia [32], renal injury-associated atherosclerosis [33] and colorectal cancer [34]. However, Wang et al. [35] indicated that miR-92a-3p could reverse the anti-tumor abilities of small nucleolar RNA host gene 14 (SNHG14) in the progression of glioma. In this experiment, the mice were treated intrathecally by agomiR-92 to investigate the functions of miR-92a-3p, and the data demonstrated that the up-regulation of miR-92a-3p observably promoted the functional recovery of the mice after SCI surgery, meanwhile, the cell apoptosis of spinal cord tissues of SCI mice was also obviously suppressed by the treatment of agomiR-92. Collectively, the improving effects of miR-92a-3p on SCI might be attributed to the inhibition of neuronal cell death.

Furthermore, our data showed that miR-92a-3p can directly regulate the expression of PTEN through specifically targeting the 3′-UTR of PTEN mRNA. The potential role of PTEN in regenerative capacities of CNS axons has been discovered by previous researches [12,36]. Knocking out or inhibiting PTEN has been reported to be able to induce the regenerative abilities of multiple neuronal populations such as corticospinal neurons, dorsal root ganglion, retinal ganglion cells and sensory neurons [37–39]. In addition, PTEN also functions as an inhibitor to AKT/mTOR pathway [40], which plays an important role in cell proliferation and survival [41,42]. In AKT/mTOR pathway, phosphoinositide 3-kinases (PI3Ks) convert phosphatidylinositol (4,5)-bisphosphate (PIP2) into phosphatidylinositol (3,4,5)-trisphosphate (PIP3), subsequently activates Akt, mTOR and ribosomal protein (S6), ultimately realizing its neuroprotective effects [43]. However, after SCI, the up-regulation of PTEN can obviously reverse the conversion of PIP2 into PIP3, leading to the inactivation of AKT/mTOR pathway [10]. We also detected the up-regulation of PTEN in the spinal cord tissues derived from the mice after SCI. Although the functions of PTEN in neuroprotection have been widely reported, the transgenic approach cannot be applied to SCI therapy in humans. Recently, miRNAs were used to control the expression of PTEN. Zhu et al. [22] demonstrated that miR-494 could directly regulate the expression of PTEN, and up-regulating the level of miR-494 in SCI rats effectively down-regulates the mRNA and protein levels of PTEN and then promotes the activation of AKT/mTOR pathway. Similarly, miR-29a has also been identified to be able to specifically target the 3′-UTR of PTEN mRNA, and overexpressed miR-29a by a recombinant lentiviral vector greatly enhances the phosphorylation of Akt and S6 and promotes the functional recovery of hindlimbs of SCI rats [44]. These studies suggest that miRNAs targeting PTEN is possibly a promising and effective strategy to treat SCI. In our study, dual-luciferase reporter assay showed that miR-92a-3p could also directly reduce the expression of PTEN. In addition, the protein levels of p-Akt and p-mTOR were obviously up-regulated by the treatment of agomiR-92. Therefore, the protective effects of miR-92a-3p after SCI might be mediated through the activation of the PTEN/AKT/mTOR pathway.

To conclude, miR-92a-3p could promote motor functional recovery and effectively inhibits cell apoptosis of spinal cord cells in SCI mice. PTEN is identified as a target for miR-92a-3p. Furthermore, enhancing miR-92a-3p increases the phosphorylation levels of Akt and mTOR after SCI. Considering the association between PTEN and AKT/mTOR pathway, our data suggest that the neuroprotective abilities of miR-92a-3p in SCI are highly associated with the activation of the PTEN/AKT/mTOR pathway.

## Abbreviations

agomiR-92: miR-92a-3p agomiR
AKT: protein kinase B
BBB: Basso, Beattie, and Bresnahan
Bcl-2: B-cell lymphoma-2
CNS: central nervous system
C caspase-3: cleaved caspase-3
GAPDH: glyceraldehyde-3-phosphate dehydrogenase
HE: Hematoxylin and eosin staining
mTOR: mammalian target of rapamycin
NC: negative control
PIP2: phosphatidylinositol (4,5)-bisphosphate
PIP3: phosphatidylinositol (3,4,5)-trisphosphate
PTEN: phosphatase and tensin homolog
SCI: spinal cord injury
